# Differential Mechanism of Periodontitis Progression in Postmenopause

**DOI:** 10.3389/fphys.2018.01098

**Published:** 2018-08-14

**Authors:** Dong-Joon Lee, Lei Wu, Masaki Shimono, Zhengguo Piao, David W. Green, Jong-Min Lee, Han-Sung Jung

**Affiliations:** ^1^Division in Anatomy and Developmental Biology, Department of Oral Biology, Oral Science Research Center, BK21 PLUS Project, Yonsei University College of Dentistry, Seoul, South Korea; ^2^Key Laboratory of Oral Medicine, Guangzhou Institute of Oral Disease, Stomatological Hospital of Guangzhou Medical University, Guangzhou, China; ^3^Department of Pathology, Tokyo Dental College, Chiba, Japan; ^4^Applied Oral Biosciences, Faculty of Dentistry, The University of Hong Kong, Hong Kong, Hong Kong

**Keywords:** periodontitis, lipopolysaccharide, ovariectomy, estrogen deficiency, immune cell migration

## Abstract

Over the past four decades, it has become accepted that periodontal disease is caused by specific bacterial infections and that individuals are uniformly susceptible neither to these infections nor to the damage caused by them. The specific bacterial infections and the composition of the environment in which these bacteria easily settle cause an immune response. The immune cells involved in pathogenesis of periodontitis migrate into the periodontitis lesion and advance the disease. The purpose of the present study is to investigate the correlation between immune cell migration and progression of periodontal disease by inducing estrogen deficiency through ovariectomy (OVX) to mimic postmenopausal women and treatment with lipopolysaccharide (LPS). The LPS derived from *Porphyromonas gingivalis* induced periodontitis and absorption of the alveolar bone dose-dependently. However, the alveolar crest level reduction after LPS injection between OVX and Sham operated mice did not show a significant difference. Matrix metallopeptidase-9 (MMP-9), which is known to be able to detect the progression of periodontitis in general, was not significantly different between OVX and Sham groups. However, immune cells such as T-lymphocytes and neutrophils migrated less overall in OVX groups than Sham operated groups. These findings can be a topic of debate on the old controversy regarding the relationship between periodontal disease and hormonal change. Currently, in clinical practice, menopause is not a major consideration in the treatment of periodontal disease. This study suggests that treatment methods and medication should be considered in the treatment of infectious periodontal disease in postmenopausal women.

## Introduction

The incidence and progression of periodontitis vary by general health, age, and sex (Holtfreter et al., 2015; Loos et al., 2015). The causes of periodontal diseases in textbooks are classified into environmental factors such as smoking, local factors like oral bacterial flora, and systemic factors. The systemic factors are nutritional status, hematologic disorder and hormonal abnormality (Petersen and Ogawa, 2012; Reynolds, 2014). During the past four decades, it has become accepted that periodontal disease is caused by specific bacterial infections and that individuals are uniformly susceptible neither to these infections nor to the damage caused by them (Van Dyke and Sheilesh, 2005). The specific bacterial infections and the composition of the environment in which these bacteria easily settle cause an immune response. Immune cells involved in the pathogenesis of periodontitis migrate into the periodontitis lesion and advance the disease (Schmidt et al., 2014; Meyle and Chapple, 2015). The cytokines from these cells cause an inflammatory response and alveolar bone resorption (Lam et al., 2014; Hao et al., 2015).

The inflammatory response varies among individuals. In addition, whether hormonal changes affect periodontal disease is controversial. It has been known that periodontitis is more common in postmenopausal women, which is due to hormonal changes, and in patients suffering from osteoporosis (Martinez-Maestre et al., 2010; Esfahanian et al., 2012; Guiglia et al., 2013). However, some research groups insist that there is a scarce relationship between menopause and periodontal disease (Kuo et al., 2008; Dvorak et al., 2011; Alves et al., 2015).

Despite the debate between menopause and periodontal disease, the past studies on periodontal diseases mainly focused on the pathogenesis of periodontal disease, while the prospective studies focused on the effect of age or sex on periodontal disease. However, the mechanism of periodontitis in postmenopausal women and young women has not been elucidated.

The purpose of the present study is to investigate the correlation between immune cell migration and progression of periodontal disease in postmenopausal women by inducing estrogen deficiency through ovariectomy (OVX) to mimic postmenopausal women and treatment with lipopolysaccharide (LPS). OVX can induce bone loss due to estrogen deficiency in rodents (Kalu, 1991; Bouxsein et al., 2005). This OVX-induced bone loss also occurs in the alveolar bone, and periodontal disease can exacerbate systemic osteopenia in OVX treated mice (Anbinder et al., 2016). The estrogen deficiency causes a decline of bone metabolism in both male and female of human (Cauley, 2015). Although, the estrogen therapy as the treatment for the deficiency is more effective in women than men (Weitzmann and Pacifici, 2006). For these reasons, the present study is confined to female in which the estrogen deficiency model could be easily made and the effect of estrogen deficiency could be seen more clearly.

LPS is an endotoxin found in the outer membrane of Gram-negative bacteria and is known to elicit strong immune responses in animals (Abbas and Lichtman, 2006). It is known that the injection of LPS derived from several species of specific bacteria causes inflammatory alveolar bone loss (Sartori et al., 2009; Graves et al., 2012; Park et al., 2013).

In the present study, the LPS derived from *Porphyromonas gingivalis* (PG) induced periodontitis and absorption of the alveolar bone dose-dependently. However, the alveolar crest level reduction after LPS injection between OVX and Sham operated mice did not show a significant difference. Matrix metallopeptidase nine (MMP-9), which is one of markers known to be able to detect the progression of periodontitis in general (Mäkelä et al., 1994; Beikler et al., 2008), was not significantly different between the OVX and Sham groups. Differently, immune cells such as T-lymphocytes (T cells) and neutrophils migrated less overall in the OVX group than the Sham operated group. With these results, we analyzed the relationship between immune response/inflammation progression and the hormonal change by OVX.

The primary aim of this study was to investigate the correlation between immune cell migration and progression of periodontal disease; and secondary, to provide evidence to solve this old debate. Through the results of the present study, the following posteriori hypothesis was derived; Estrogen deficiency may lead to periodontitis through adaptive immune response rather than innate immune response.

## Materials and Methods

All animal experiments were approved by Yonsei University Health System Institutional Animal Care and Use Committee (YUHS-IACUC) in accordance with the Guide for the care and use of laboratory animal (National Research Council, United States). The animal study plan for these experiments (2016–0342) was reviewed and approved by this committee on January 28, 2017. And all experiments were performed in accordance with the guidelines of this committee.

### Animals Model Preparation

C57BL/6 8-week-old female mice were purchased from KOATECH (Pyeongtaek, Korea) and randomly divided into two groups (*n* = 10 per group). All animals were housed in a temperature-controlled room (22°C) under artificial illumination with a 12-h light/dark cycle and 55% relative humidity. The mice were provided access to food and water *ad libitum*. All operational procedures were performed under deep anesthesia. OVX was performed in one group (OVX group) of 8-week-old mice. Incisions and sutures on the back of mice were performed in the other group (Sham group).

### Inflammatory Bone Loss Model

**FIGURE 1:**
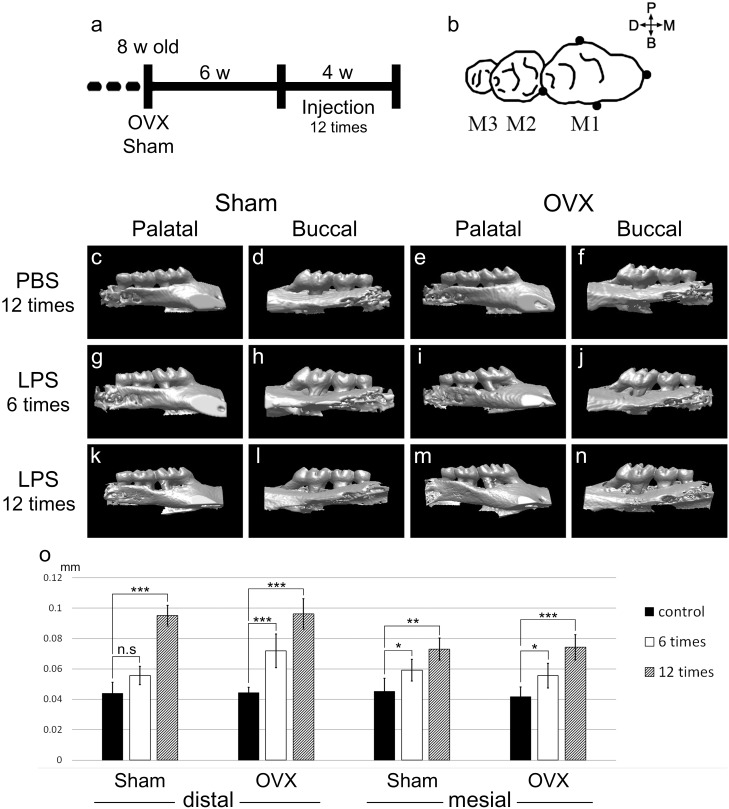
LPS application and alveolar bone crest level reduction. **(a)** Schematic diagram depicting timing of the experimental design. OVX or Sham was operated in 8-week-old mice. Six weeks after the operation, PBS and LPS were injected 12 times for 4 weeks. **(b)** The four injection points (black dots) around maxillary M1. 5.0 μg of LPS in 2.5 μl of PBS or 2.5 μl of PBS was injected per point in an injection time. **(c–n)** Micro-CT 3D reconstructed images captured from the palatal or buccal side. **(c–f)** CT images were scanned at 3 days after the 12th injection of PBS for the control groups. **(g–j)** At 6th injection period. **(k–n)** At 3 days after 12th injection period. 3D images of the LPS groups **(g–n)** were flipped left and right for directional unification. **(o)** CEJ-ABC distances were measured in the mesial and distal side of M1 from CT scan images. The results are expressed as the mean ± *SD* (*n* = 15, three CT images per group of five mice). ^∗^*p* < 0.05, ^∗∗^*p* < 0.01, ^∗∗∗^*p* < 0.005.

The bone loss model was generated by modifying the protocol previously described (Sartori et al., 2009). To initiate alveolar bone loss, we micro-injected *P.* *gingivalis* LPS (20 μg per injection time in 10 μl of PBS total volume) directly into the four points (5.0 μg per point in 2.5 μl of PBS) around the palatal left first molars (M1) of each five mice from Sham and OVX groups (**Figure 1b**). The same volume of PBS was injected into the same points on the right side. LPS and PBS injections were repeated three times each week for 4 weeks. Three days after last injection time, all animals were sacrificed by carbon dioxide asphyxiation.

### Micro-Computed Tomography (CT) and Alveolar Bone Crest Level Analysis

Micro-CT (CT) analysis was performed as previously described (Gully et al., 2014). Mice were scanned using the SkyScan 1076 High Resolution live animal computed tomography (SkyScan, Bruker, Belgium) to determine changes in bone volume in the cemento-enamel junction to the alveolar bone crest (CEJ-ABC) length in the maxillae. Animals were anesthetized prior to scanning, positioned on a polystyrene foam holder, and then placed in the CT scanner. Mice were scanned at the 6th injection period (2 weeks after the first injection time) and again at the completion of the study (3 days after last injection).

To realign image data the appropriate plane for determining the CEJ-ABC distance, DataViewer (Version 1.4.4) was used. Image data were realigned as the midline passing M1 to palatal left third molar (M3) would be the sagittal plane. Then sagittal sectional images were saved for the CEJ-ABC distance. The images were opened in CT Analyser (Version 1.12.0.0) to measure the CEJ-ABC distance. The CEJ line of M1 was set manually in the sagittal CT image and measured the distance from CEJ line to the ABC on the mesial and distal sides of M1 (**Supplementary Figure 1**). Three parallel serial sagittal images were used to measure CEJ-ABC for each mouse.

Scans were three-dimensionally reconstructed (3D images) using SkyScan NRECON software (Version 1.6.6.0).

### Immunohistochemistry (IHC)

After the CT scan, maxillae were hemisected, and each tissue was immersed in 4% paraformaldehyde for up to 24 h. After fixation, the tissues were decalcified in 10% sodium citrate and 22.5% formic acid for 6 weeks at 4°C. Staining was performed on 6 μm paraffin-embedded sections. After deparaffinization, the slides were incubated with pepsin (Digest-All^TM^ 00–3009, Invitrogen, United States) for 10 min at 37°C. Subsequently, the slides were incubated with antibodies against MMP-9 (1:100 diluted, AB19016, EMD Millipore Co., United States), myeloperoxidase (MPO, 1:200 diluted, Rb-373-A0, Thermo Fisher Scientific, United States) or CD3 (1:100 diluted, ab5690, Abcam plc, United Kingdom) at 4°C overnight. The specimens were sequentially incubated with secondary antibodies and streptavidin peroxidase. The results were visualized following staining with a diaminobenzidine (DAB) reagent kit (Invitrogen, United States). The sections were counterstained with Mayer’s hematoxylin. All specimens were observed using a stereomicroscope (MD5500D; Leica, camera: DFC495; Leica, Lens: HCX PL APO 409; Leica).

### Statistics

CEJ-ABC distance data were expressed as the mean ± SD from three slices of CT images for each mouse. For counting the number of cells, three squares (0.2 mm × 0.2 mm) were randomly selected from two cross-sectional images taken at the distal and mesial sides of M1s for five mice of each group (see **Supplementary Figure 2**). Totally, nine sectional images from five mice of each group were used for one target protein of IHC. The cell numbers were counted in 27 squares (nine images × 3 squares) of each group (distal and mesial side, respectively). All the positive and negative cells in the squares were counted manually. The number of positive cells to the total cell number was expressed as a percentage. Statistical analysis was performed by one-way analysis of variance (ANOVA) followed by Student’s *t*-test. For all analyses, *p* < 0.05 was considered significant.

**FIGURE 2:**
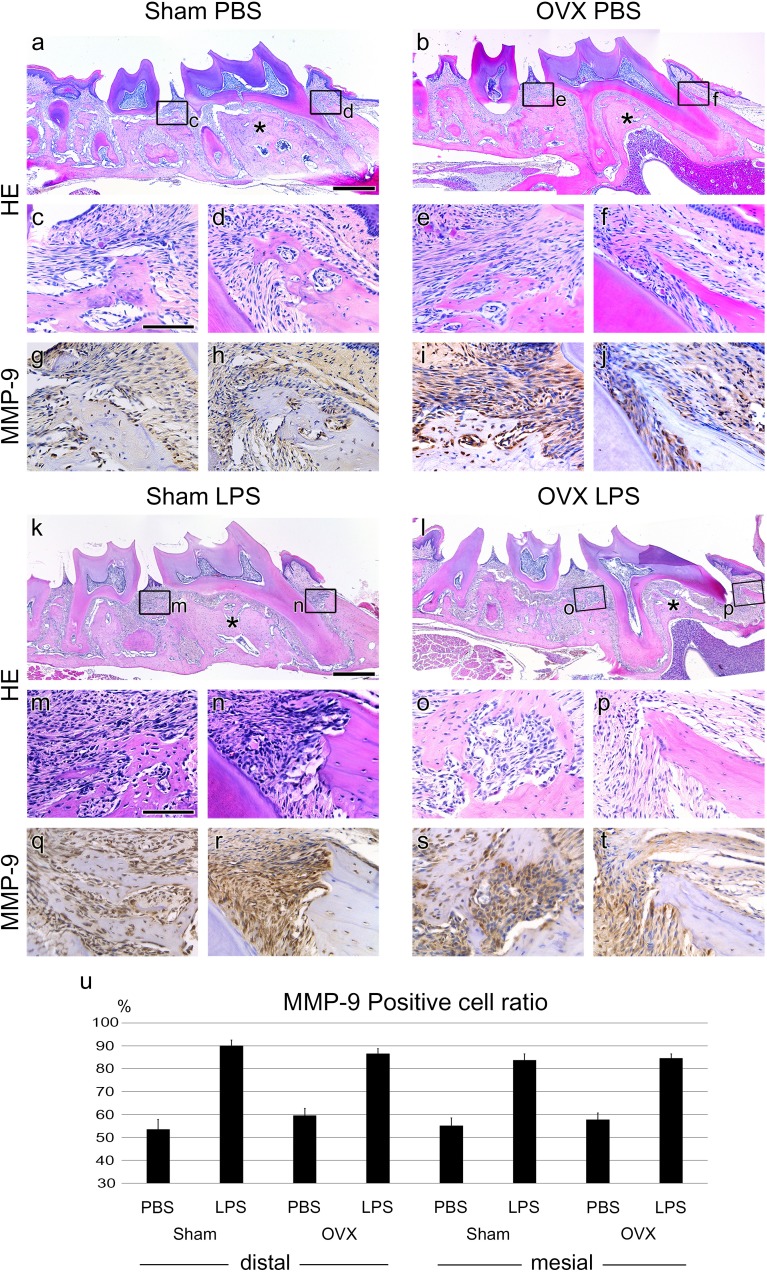
Periodontitis progression of Sham and OVX mice. **(a,b,k,l)** Sagittally sectioned HE-stained image of PBS or LPS injected in Sham and OVX mice. Asterisks (^∗^) indicate inter-radicular area of M1. **(c–f, m–p)** High magnification of panel **a**,**b**,**k,l**. **(d,f,n,p)** Mesial side of M1. Alveolar bone thicknesses are different between Sham and OVX groups. **(g–j, q–t)**. IHC of the periodontitis progression marker, MMP-9. The MMP-9-positive cells were around the PDL tissue of the injected position. **(n)** The ratio of MMP-9-positive cells to total cells. The results are expressed as the mean ± *SD* (*N* > 25, three 0.04 mm^2^ squares were randomly chosen from the two section images for each mouse). MMP-9-positive cells between PBS and LPS treated samples of all groups were significant (*p* < 0.005). Scale bars, **a**,**b**,**k**,**l**, 500 μm; **c–j, m–t**, 100 μm.

## Results

### LPS Application and Alveolar Bone Crest Level Reduction

To mimic the body after menopause in mice, OVX was performed. Eight-week-old female mice were ovariectomized or Sham operated as a control. Six weeks after operation, to induce periodontitis in the mouse maxillary first molar, LPS or Phosphate Buffered Saline (PBS) was micro-injected 12 times over 4 weeks (**Figure 1a**). LPS or PBS was injected in four points around M1 in one injection time (**Figure 1b**).

The effects of LPS injection were assessed by CT images. The CT results of each experimental specimen were reconstructed to 3D images and captured from the palatal side and buccal side (**Figures 1c–n**). In the Sham control group (PBS 12 times treated), the mean CEJ-ABC distances of five mice were 44.08 μm in the distal and 45.24 μm in the mesial side. In the Sham LPS injected group, the CEJ-ABC distances were significantly increased compared to the control group, and the ABC level was lowered at the 12th treatment (distal; 95.12 μm, mesial; 73.08 μm) compared to the 6th treatment (distal; 55.68 μm, mesial; 59.16 μm), with significant differences in mean values (**Figure 1o**). The OVX groups showed a similar pattern to the Sham groups. The mean CEJ-ABC distances of the OVX control group were 44.31 μm in the distal and 41.76 μm in the mesial side. Those of the OVX LPS group were distal; 71.92 μm, mesial; 55.68 μm at 6th injection, and distal; 96.28 μm, mesial; 74.24 μm at 12th injection. However, there was no significant difference between the OVX and Sham groups.

### Periodontitis Progression of Sham and OVX Mice

To analyze periodontitis progression histologically, Hematoxylin and Eosin (HE) staining and IHC against MMP-9 were performed. The volumes of the alveolar bone in the OVX groups were reduced in the inter-radicular area of M1 compared to the Sham groups (**Figures 2a,b,k,l** asterisks). The thicknesses of the mesial side alveolar bone of M1 were also lower in the OVX groups than in the Sham groups (**Figures 2d,f,n,p**). In the LPS groups, the scalloped borders were shown around the injection area (**Figures 2m–p**) but not in the PBS-injected control groups (**Figures 2c–f**).

In the Sham groups, LPS injection showed presence of granulocytes and increased numbers of lymphocytes usually shown in the typical chronic inflammation tissue (**Figures 2c,d,m,n**). However, in the OVX groups, the increase in chronic inflammatory cells by LPS injection was not as high as in the Sham groups (**Figures 2e,f,o,p**).

The expression of MMP-9 as a periodontitis marker was significantly increased in the LPS groups compared to the PBS groups (**Figures 2g–j, q–t**). The number of MMP-9 expressing cells was counted, and the ratio of MMP-9 positive cells to the total cells was calculated (**Figure 2u**). A significant difference between LPS and PBS groups was shown, but not between the Sham and OVX groups.

### Immune Cell Migration

**FIGURE 3:**
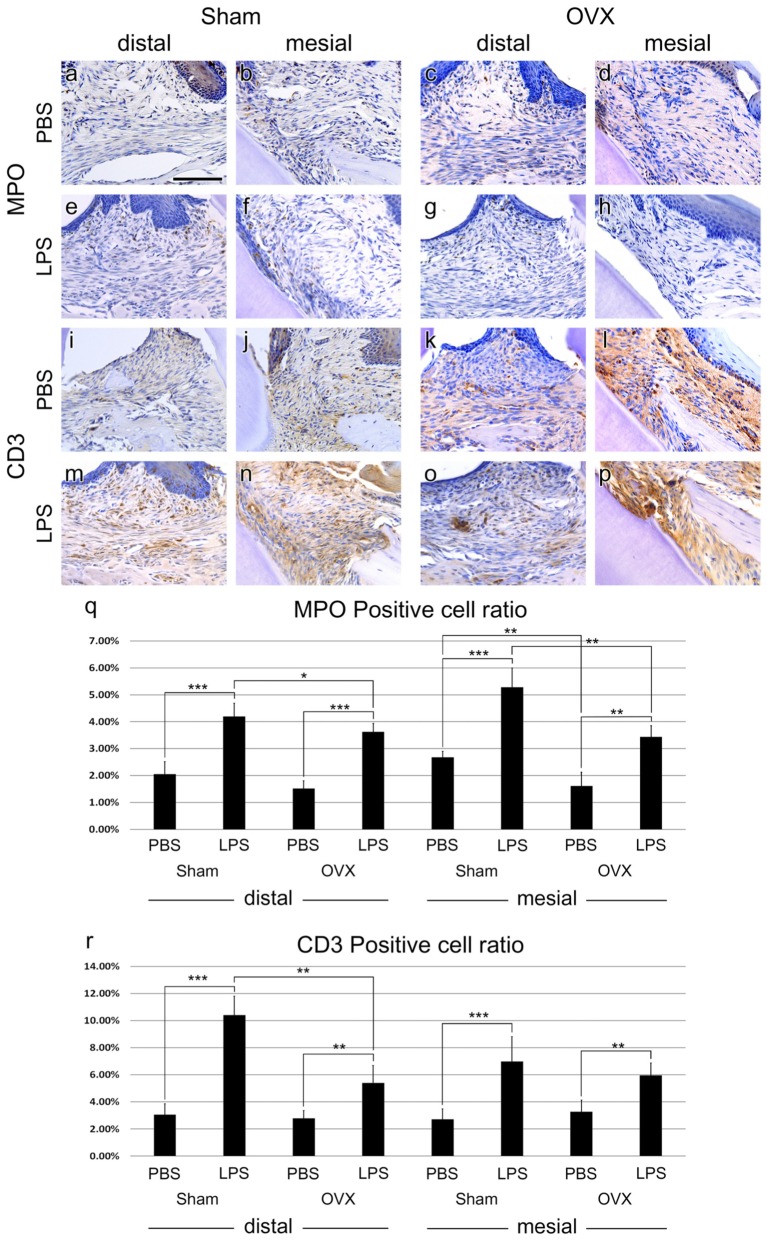
Immune cell migration. **(a–h)** IHC of neutrophil marker, MPO. MPO-positive cells were found just below the epithelium of the injection area. **(i–p)**, IHC of T cell marker, CD3. CD3-positive cells were shown all around the injection area. **(q,r)** MPO- and CD3-positive cells ratio analysis at the injection area. The results are expressed as the mean ± SD. ^∗^*p* < 0.05, ^∗∗^*p* < 0.01, ^∗∗∗^*p* < 0.005. Scale bar, 100 m.

To investigate the immune responses in the Sham and OVX groups, IHC against neutrophil and T-lymphocyte was performed. MPO positive neutrophils were distributed in the connective tissue and some in the epithelium of the PBS-injected groups (**Figures 3a–d**). In the LPS groups, the numbers of neutrophils were increased compared to the Sham groups (**Figure 3q**). Neutrophils were collected just below the epithelium of the injection area (**Figures 3e–h**). Between the Sham and OVX groups, neutrophil migration into the lesion showed a difference. The neutrophil migration was decreased in the OVX groups significantly, regardless of LPS injection (**Figure 3q**).

In both PBS and LPS groups, CD3 positive T cells were distributed all around the injection areas. Like MPO positive cell ratio, T-cell migration was increased by LPS injection compared to PBS injection (**Figure 3r**). Specifically, in the distal side of M1 of the Sham group, T-cell migration was prominent (**Figures 3i,m**). Additionally, T-cell migration between the OVX and Sham groups was significantly different in LPS treatment at the distal side (**Figures 3m,o**). However, T-cell migration was not significant in PBS treated groups (**Figures 3i–l**) and in mesial sides of LPS treated groups (**Figures 3n,p**). The exact positive cell ratio values are summarized in **Table 1**.

**Table 1:** Numerical analysis of immune cell infiltration between Sham and OVX groups.

		Unit: %			
		Distal		Mesial	
		Neutrophil	T cell	Neutrophil	T cell
Sham	PBS (*n* = 27)	2.05 ± 0.47	3.06 ± 0.81	2.67 + 0.23	2.72 ± 0.76
	LPS (*n* = 27)	4.19 ± 0.50	10.40 ± 1.41	5.28 + 0.72	6.98 ± 1.84
	*p*-value	0.004	0.002	0.004	0.02

OVX	PBS (*n* = 27)	1.52 ± 0.29	2.79 ± 0.57	1.61 ± 0.51	3.27 ± 0.85
	LPS (*n* = 27)	2.82 ± 0.32	5.38 ± 1.31	3.43 ± 0.43	5.94 ± 0.93
	*p*-value	0.004	0.018	0.008	0.005

LPS	Sham (*n* = 27)	4.19 ± 0.50	10.40 ± 1.41	5.28 + 0.72	6.98 ± 1.84
	OVX (*n* = 27)	2.82 ± 0.32	5.38 ± 1.31	3.43 ± 0.43	5.94 ± 0.93
	*p*-value	0.03	0.021	0.012	n.s

The ratios of the immune cells to total cells are displayed in this table. First, the data were compared by PBS and LPS groups. In the LPS groups, the number of immune cells was increased in both the Sham and OVX groups. The degree of increase was different. Specifically, LPS treated groups were compared to the Sham and OVX groups. It is evident that the immune cell infiltration occurs less in the OVX group. The sample size (*n*) means the number of cell-counted squares.

## Discussion

Activation of the innate immune system is critical for lymphocyte activation and other immune cells to help clear infectious micro-organisms (Sartori et al., 2009). However, the relationship between the mechanism of the immune system and hormonal change is not clear. The controversial correlation of periodontitis progression and menopause is also a task that must be revealed. To bridge these two problems, we performed OVX and LPS injection in the mice to imitate postmenopausal women suffering from periodontitis.

The distance from the CEJ to the ABC is one of the most widely used methods measuring the progression of periodontitis (Darby et al., 2005; Park et al., 2007; Liang et al., 2010). 2D CT images showed that ABC was decreased in both the mesial and distal sides dose-dependently. However, the change in ABC level after 12 injections showed no significant difference between the two groups. Only the ABC at the distal side of M1 decreased more in the OVX group at the 6th LPS injection. This suggests that in the M1 distal side, or the interproximal area of M1 and palatal left second molar (M2), LPS-induced periodontitis progresses more rapidly in the OVX group.

Histological analysis also confirmed the change around the injection points. In the LPS-injected groups, there were scalloped borders showing bone resorption and eosinophilic cells, which were considered to be macrophages forming lacunae, but not in the PBS-injected control groups.

MMP-9 is one of the proteolytic enzymes released by the host cells and is associated with tissue destruction in periodontal disease states with MMP-2 (Mäkelä et al., 1994). A previous study of profiling gene expression based on biopsy samples from patients with severe chronic periodontitis also suggested that several types of MMPs can be used as markers for the progression of periodontitis (Beikler et al., 2008). Histological analysis revealed that the number of cells expressing MMP-9 at the injection site was significantly different between the LPS groups and the control groups, which was similar to the decrease pattern of ABC level. This confirms that MMP-9 can function as a marker for the progression of periodontitis. However, the difference in the ratio of MMP-9 positive cells to the total cells in region of interest (ROI) between the Sham and OVX groups was not significant. This means that hormonal changes due to menopause and the progression of periodontitis are not significant. Furthermore, it can be evidence to support the negative correlation argument in the debate mentioned above.

Additionally, the alveolar bone volume of M1 inter-radicular areas and the thickness of M1 mesial side alveolar bone were reduced in the OVX groups compared to Sham groups. This aspect was not found between LPS groups and PBS groups. The bone volume differences in both regions of M1 were due to hormonal changes by OVX, not due to the LPS injection. However, bone volume is not a diagnostic criterion for periodontitis evaluation from clinical point of view, the attachment level (almost same as CEJ-ABC) is the most important criterion (Highfield, 2009).

We analyzed neutrophils and T cells in the lesion as representative of innate immune cells and adaptive immune cells, respectively. The analysis was carried out in two respects; Between OVX and Sham groups, between LPS and PBS groups. Neutrophils are also known to cause inflammatory reactions throughout the progression phase of periodontitis (Van Dyke, 2008; Hajishengallis and Hajishengallis, 2014). Specifically, the significantly greater abundance of neutrophils in the gingiva is the most notable cellular difference in the beginning of periodontitis (Van Dyke, 2007; Dutzan et al., 2016). This inflammatory condition induces infiltration of other circulating immune cells including T cells into lesions. In this study, MPO-positive cells were naturally found more in the LPS groups than in the PBS groups. However, the neutrophil distribution showed a different pattern between the Sham and OVX groups, compared to a lack of difference in the MMP-9 results. The neutrophils were significantly less frequent in the OVX groups, both with OVX LPS and OVX PBS, compared to the Sham groups. Previous studies have shown that low level of estrogen attenuates the inflammatory response, while this estrogen deficiency allows an infection to occur easily (Pfeilschifter et al., 2002; Straub, 2007). These studies showed the paradox of the MMP-9 level sustainment despite the reduction in neutrophil infiltration in this study, which could be resolved.

T cells can be found in the dense inflammatory infiltrate in periodontal disease (Taubman and Kawai, 2001). T cells are one of the primary sources of the receptor activator of nuclear factor kappa-B ligand (RANKL), which regulates osteoclast formation (Kawai et al., 2006). In the present study, the numbers of CD3+ T cells were deservedly increased in the LPS-injected groups compared to the PBS-injected groups. In the PBS groups, a difference in T cells infiltration between in OVX and Sham groups was not found. On the other hand, the ratio of T cells in the OVX LPS group was significantly lower than that of the Sham LPS group, like neutrophils.

The reductions in infiltration of both neutrophils and T cells in the OVX groups may be due to a decrease in the systemic immune cells of the OVX groups. This hypothesis can be supported by a previous study analyzing the composition of mononuclear cells isolated from the spleen and bone marrow of OVX-induced osteopenic mice (Garcia-Perez et al., 2006). This previous study showed that CD3+ T cells were reduced in the spleen and bone marrow of OVX mice compared to Sham-operated mice, but RANKL+/CD3+ T cells were increased in bone marrow. This implies that osteoclast precursors are differentiated into osteoclasts by the ability of the T cell to regulate osteoclast differentiation rather than the number of T cells that infiltrated into the lesion. It can be inferred that T cell-mediated adaptive immune response predominates in OVX groups.

## Conclusion

In Conclusion, OVX does not affect the degree of periodontitis progression. However, the mechanisms or progression phase of periodontitis was significantly different between the OVX and Sham groups. Our results suggest that periodontitis may progress through adaptive immune response rather than innate immune response by estrogen deficiency. This phenomenon can occur in periodontitis progression in postmenopausal women.

Furthermore, these findings can be a topic of debate of the old controversy regarding the relationship between periodontal disease and hormonal change. Currently, in clinical practice, menopause is not a major consideration in the treatment of periodontal disease. This study suggests that treatment methods and medications should be considered in the treatment of infectious periodontal disease in postmenopausal women.

## Author Contributions

D-JL, J-ML, and H-SJ conceptualized the study and designed the experiments. D-JL drafted the manuscript. D-JL and LW conducted the experiments. ZP, MS, and DG critically revised the manuscript for intellectual content. D-JL, MS, J-ML, and H-SJ analyzed and interpreted the results. All authors gave permission to the final draft of the manuscript.

## Conflict of Interest Statement

The authors declare that the research was conducted in the absence of any commercial or financial relationships that could be construed as a potential conflict of interest.
