# Supplementary material for: Differential Mechanism of Periodontitis Progression in Postmenopause
Source: Front Physiol. 2018 Aug 14;9:1098. doi: 10.3389/fphys.2018.01098 (PMC6113945; doi:10.3389/fphys.2018.01098)
Supplement: Supplementary file 1 [file Image_1.pdf]

Differential mechanism of periodontitis progression in postmenopause

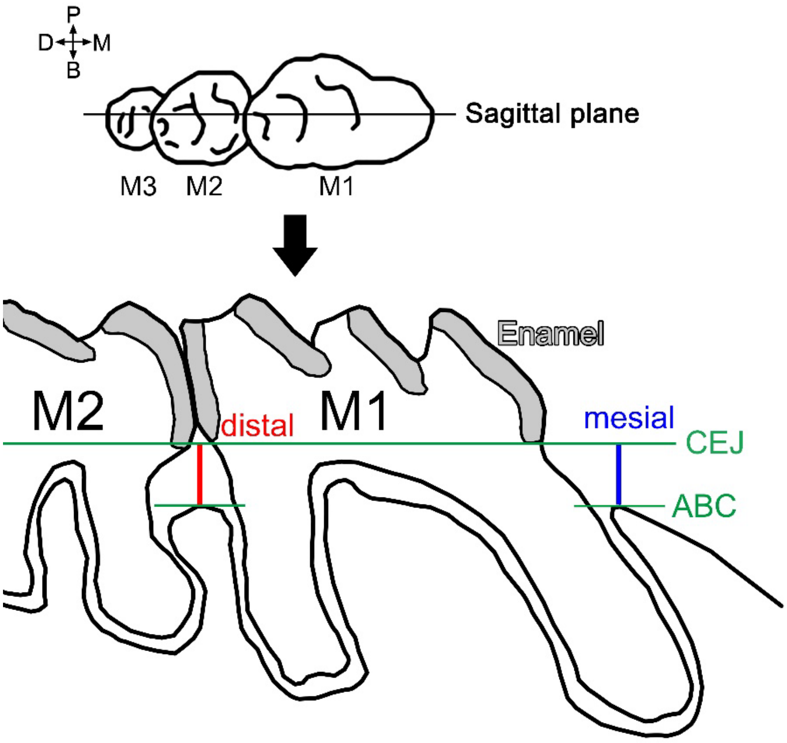

Supplementary Figure 1 | CEJ-ABC measurement from micro CT image.

CT Image data were realigned as the midline passing M1 to M3 would be the sagittal plane. The CEJ line of M1 was set manually at the mesial and distal margins of enamel in the sagittal image. CEJ-ABC distances were measured on the mesial and distal sides of M1. Three parallel serial sagittal images were used for each mouse. Outlines of teeth and alveolar bone contour were traced from sagittal CT image.
