# Supplementary material for: Differential Mechanism of Periodontitis Progression in Postmenopause
Source: Front Physiol. 2018 Aug 14;9:1098. doi: 10.3389/fphys.2018.01098 (PMC6113945; doi:10.3389/fphys.2018.01098)
Supplement: Supplementary file 2 [file Image_2.pdf]

Differential mechanism of periodontitis progression in postmenopause

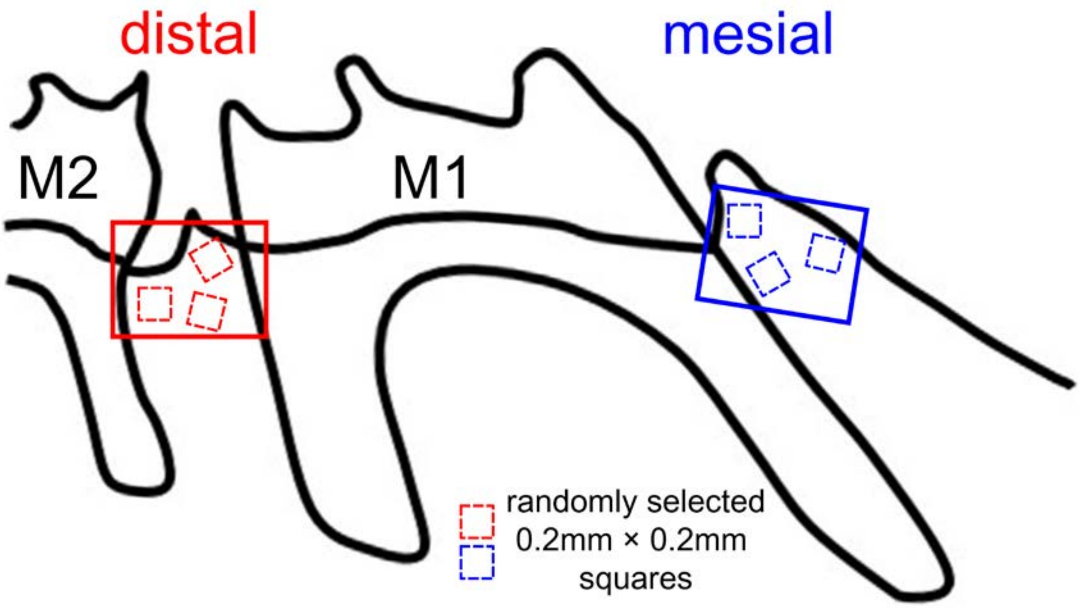

Supplementary Figure 2 | Infiltrated cell count from IHC images.

Three squares (0.2 mm × 0.2 mm) were randomly chosen from the two section images taken by stereomicroscope at distal and mesial sides of M1s from five mice of each group. The total cell number and the positive cell number were counted manually. Outlines of teeth and gingival mucosa were obtained by tracing the paraffin-embedded section image.
